# Clinical impact of methicillin-resistant *staphylococcus aureus* on bacterial pneumonia: cultivation and 16S ribosomal RNA gene analysis of bronchoalveolar lavage fluid

**DOI:** 10.1186/s12879-016-1493-3

**Published:** 2016-04-16

**Authors:** Toshinori Kawanami, Kazuhiro Yatera, Kei Yamasaki, Shingo Noguchi, Kazumasa Fukuda, Kentarou Akata, Keisuke Naito, Takashi Kido, Hiroshi Ishimoto, Hatsumi Taniguchi, Hiroshi Mukae

**Affiliations:** Department of Respiratory Medicine, University of Occupational and Environmental Health, 1-1 Iseigaoka, Yahatanishiku, Kitakyushu city, Fukuoka 807-8555 Japan; Department of Microbiology, University of Occupational and Environmental Health, 1-1 Iseigaoka, Yahatanishiku, Kitakyushu city, Fukuoka 807-8555 Japan

**Keywords:** 16S rRNA gene, Methicillin-resistant *Staphylococcus aureus,* MRSA, Clone library, Contamination, Pneumonia, Bronchoalveolar lavage, BALF, Pneumonia

## Abstract

**Background:**

Determining whether methicillin-resistant *Staphylococcus aureus* (MRSA) is a true causative pathogen or reflective of colonization when MRSA is cultured from the respiratory tract remains important in treating patients with pneumonia.

**Methods:**

We evaluated the bacterial microbiota in bronchoalveolar lavage fluid (BALF) using the clone library method with a 16S ribosomal RNA (rRNA) gene analysis in 42 patients from a pneumonia registry who had MRSA cultured from their sputum or BALF samples. Patients were divided into two groups: those treated with (Group A) or without (Group B) anti-MRSA agents, and their clinical features were compared.

**Results:**

Among 248 patients with pneumonia, 42 patients who had MRSA cultured from the respiratory tract were analyzed (Group A: 13 patients, Group B: 29 patients). No clones of *S. aureus* were detected in the BALF of 20 out of 42 patients. Twenty-eight of 29 patients in Group B showed favorable clinical outcomes, indicating that these patients had non-MRSA pneumonia. Using a microflora analysis of the BALF, the *S. aureus* phylotype was predominant in 5 of 28 (17.9 %) patients among the detected bacterial phylotypes, but a minor population (the percentage of clones ≤ 10 %) in 19 (67.9 %) of 28 patients. A statistical analysis revealed no positive relationship between the percentage of clones of the *S. aureus* phylotype and risk factors of MRSA pneumonia.

**Conclusions:**

The molecular method using BALF specimens suggests that conventional cultivation method results may mislead true causative pathogens, especially in patients with MRSA pneumonia. Further studies are necessary to elucidate these clinically important issues.

## Background

Patients with nosocomial pneumonia caused by methicillin-resistant *Staphylococcus aureus* (MRSA) have been increasing over the past half century. Approximately 20–40 % of all hospital-acquired pneumonia (HAP), ventilator-associated pneumonia (VAP) [1, 2] and the number of MRSA pneumonia patients is increasing in step with aging of the population [3]. Several guidelines [4, 5], including Japanese guidelines for nursing and healthcare-associated pneumonia (NHCAP) [6] and HAP [7, 8], suggest the use of anti-MRSA antimicrobials in pneumonia patients when the risks of MRSA are suggested. However, there have been only a few clinical studies that describe the pathogenicity of MRSA in bacterial pneumonia and accurate diagnostic methods for evaluating MRSA pneumonia [9–11]. Generally, the diagnostic criteria of respiratory infection caused by MRSA are positive results of a quantitative culture of MRSA over 10^6^ colony forming units (CFU)/ml in sputum samples, 10^4^ CFU/ml in lower respiratory specimens and/or phagocytosis of *S. aureus* by polymorphonuclear neutrophils [4]. However, it is occasionally difficult to differentiate whether the detected MRSA is a true causative pathogen of pneumonia or only reflective of colonization when MRSA is cultured from the lower respiratory tract samples. Physicians should carefully consider whether or not cultured MRSA is actually causative in each case because many patients fulfill these criteria and improve without anti-MRSA agents in real-world clinical settings. Differentiation of MRSA as a cause of pneumonia or merely colonization remains an important clinical issue and is of a particular interest in clinical settings.

We hypothesized that the percentage of *S. aureus* clones in bronchoalveolar lavage fluid (BALF) directly obtained from the affected lesions of pneumonia identified by chest CT might be helpful to distinguish true MRSA pneumonia from colonization of MRSA. In the present study, we used the data from the pneumonia registry, which included 16S ribosomal RNA gene analyses of BALF, and patients with pneumonia in whom MRSA was cultured from the respiratory samples were enrolled. Then we divided these patients into two groups: Group A included MRSA pneumonia patients treated with anti-MRSA agents and Group B were patients with MRSA cultured from respiratory samples but who improved without anti-MRSA treatment, and the clinical features of these two groups were compared.

## Methods

### Patients

**Fig. 1:**
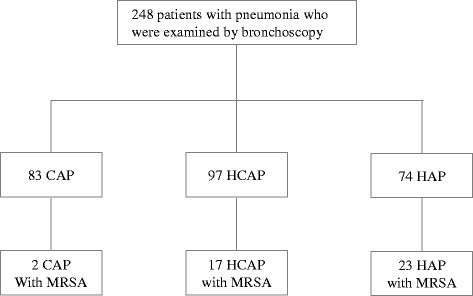
A flow chart of the study participants. CAP: community-acquired pneumonia, HCAP: healthcare-associated pneumonia, HAP: hospital-acquired pneumonia, MRSA: methicillin-resistant *Staphylococcus aureus*, BALF: bronchoalveolar lavage fluid

Among 248 Japanese patients with community-acquired pneumonia (CAP), healthcare-associated pneumonia (HCAP) and HAP at the University of Occupational and Environmental Health, Japan and referred hospitals between April 2010 and January 2015, 42 patients with positive cultures for MRSA from respiratory specimens (i.e., sputum, endobronchial aspirates and BALF) were enrolled (Fig. 1). This cohort included patients in previous studies of CAP [12] and HCAP [13]. Patients who had MRSA positively cultured from respiratory specimens were divided into two groups: Group A consisted of patients that had been treated with anti-MRSA agents, and Group B included patients that had been treated without anti-MRSA agents, and the clinical features of these two groups were compared. This study was approved by the Human and Animal Ethics Review Committee of the University of Occupational and Environmental Health, Japan (No.09-118), and all patients provided their written informed consent.

### Definitions

The diagnosis of pneumonia was made by the fulfillment of the following three criteria: (1) at least one of the following clinical symptoms (a fever ≥ 37 °C, cough, purulent sputum, moist rales, pleural pain, dyspnea, or tachypnea); (2) new infiltrates on a chest X-ray or computed tomography (CT); and (3) at least one sign of systemic inflammation (a white blood cell (WBC) count > 10,000/mm^3^ or < 4,500/mm^3^ or an increased C-reactive protein (CRP) level). The definitions of CAP, HCAP and HAP were made according to the Infectious Diseases Society of America (IDSA)/American Thoracic Society (ATS) guidelines. Briefly, CAP was defined as pneumonia acquired in the community with no risk factors for HCAP. HCAP was defined as pneumonia acquired in the community with one or more of the following risk factors: (a) hospitalization for 2 days in the preceding 90 days; (b) residence in a nursing home or extended care facility; (c) home infusion therapy (including antibiotics); (d) long-term dialysis (including hemodialysis and peritoneal dialysis) within 30 days of entering the study; and (e) home wound care. HAP was defined as pneumonia acquired in the hospital ≥ 48 h after admission [4, 14]. The criteria for aspiration risk factors, such as neurologic dysphagia, anatomical abnormalities of the upper aerodigestive tract and poor oral hygiene defined by Marik et al. [15] were used, and patients with gastroesophageal disorders (including disruption of the gastroesophageal junction) were included in this study.

### Data and sample collection

The laboratory findings and radiological information on chest X-rays and/or CT were collected. BALF specimens using 40 ml of sterile saline were obtained from lung lesions of pneumonia, as previously described [12, 13].

### Evaluation of the clinical efficacy

The clinical efficacies of the antimicrobials were evaluated by an improvement in the clinical symptoms, laboratory and chest radiography findings, which fulfill the definitions proposed by the Japan Society of Chemotherapy [16]. The treatment medication for pneumonia was considered to be clinically “effective” when more than three of the following criteria were satisfied: (1) improvement or complete resolution of the clinical symptoms, (2) improvement in the body temperature to ≤ 37 °C, (3) chest radiography score of ≤ 70 % of the previous value, (4) WBC count ≤ 9,000/mm^3^ and (5) CRP level ≤ 30 % of the previous value. When the efficacy criteria were not satisfied for any reason, the case was considered to be “ineffective.” Physicians followed the guidelines for CAP, HCAP and HAP to use anti-MRSA agents as the first antimicrobial treatment. As the present study was a retrospective cohort study, there were no strict criteria that regulate an intervention of anti-MRSA therapy as an additive antibiotic treatment, but clinical response to antibiotics was firstly evaluated three days after the start of antimicrobial treatment, physicians decided to add anti-MRSA agents when the clinical response to antimicrobials were ineffective with positive culture results for MRSA.

### Criteria for the identification of bacterial isolates

#### Microbiological evaluation using cultivation methods

Cultivation of BALF and sputum samples was performed as previously described [12, 13]. The samples were inoculated onto the appropriate Vitek apparatus with or without the associated API identification strip using the Vitek 2 apparatus (bioMerieux), and bacterial identification was confirmed according to an identification percentage of more than or equal to 90 %. When the percentage was less than 90 %, subsequent bacterial identification was performed using API. Each macroscopically recognized bacterial colony containing normal bacterial flora was recorded as normal flora. All *S. aureus* isolates were identified according to a morphologic analysis of the bacterial colony, Gram staining and catalase and coagulase tests. MRSA was identified if the minimum inhibitory concentration of oxacillin was ≥ 4 μg/ml.

#### Total cell count, cell lysis efficiency analysis and bacterial identification using the molecular method

Using BALF specimens, the total bacterial cell count and cell lysis efficiency were evaluated using epifluorescent microscopy, and DNA extraction, amplification of the 16S rRNA gene using polymerase chain reaction (PCR), clone library construction and a sequencing analysis were performed, as previously described [12, 13, 17–20]. Detected DNA sequences were then compared with those of the type strains using the basic local alignment search tool (BLAST) algorithm, as described previously. The 16S rRNA gene sequences of type strains were obtained from the DNA Data Bank of Japan (http://www.ddbj.nig.ac.jp/) and the Ribosomal Database Project II (http://rdp.cme.msu.edu/) [12, 13, 17–20].

### Evaluation of the radiologic findings

Chest X-rays or CT performed within 48 h of the onset of pneumonia were analyzed and evaluated by two experienced respirologists without any clinical information. Radiological findings were sorted into four different patterns as follows: “lobar pneumonia pattern” that showed an air-space consolidation limited to one lobe or one segment, “aspiration pneumonia pattern” that showed pulmonary infiltrations in the bilateral lower lobes, “pulmonary abscess pattern” where the infiltrations accompanied the cavity and “others” that included any other remaining patterns of pneumonia, such as bronchopneumonia, according to previous reports [21, 22].

### Statistical analysis

The baseline characteristics were summarized using descriptive statistics. Continuous variables were compared using the Mann–Whitney *U*-test and Student’s t-tests, while categorical variables were compared using Fisher’s exact test or the chi-square test, as appropriate. The SPSS software package (version 19) was used for the statistical analysis, and a *P* <0.05 was considered statistically significant.

## Results

### Clinical characteristics and laboratory findings of the participants

The clinical characteristics and laboratory findings of 42 patients from whom MRSA was detected are shown in Table 1. Thirteen (31.0 %) and 29 (69.0 %) patients had been treated with (Group A) and without (Group B) anti-MRSA antimicrobials. Between Groups A and B, the rate of patients with malignancy was significantly higher in Group B (37.9 %, 11/29) than in Group A (7.7 %, 1/13) (*p* = 0.016) (Table 1). In these 42 patients, the risk factors of MRSA, such as the use of corticosteroid or immunosuppressants (14.3 %), nasogastric tube feeding or percutaneous gastrostomy tube feeding (7.1 %), antibiotics use within 90 days (28.6 %), detection of MRSA within 90 days (23.1 %), were observed, however, these findings were not significantly different between the groups (Table 1). In addition, patients with aspiration risks were observed in 54.8 % of the total cohort. Group B included significantly more HCAP patients than Group A (*P* = 0.015), however, the radiological findings were not significantly different between Groups A and B (Table 1).

**Table 1:** The clinical characteristics and laboratory findings of 42 patients treated with or without anti-MRSA drugs in this study

Clinical variates	Group A	Group B	*P*-value
	Treated with anti-MRSA drugs	Treated without anti-MRSA drugs	
	(*n* = 13)	(*n* = 29)	
Age ± SD (years)	74.8 ± 10.1	74.6 ± 17.5	0.764
Male: Female	10:3	19:10	0.458
Underlying diseases**			
None	2 (15.4 %)	4 (13.8 %)	0.898
Malignancies	1 (7.7 %)	11 (37.9 %)	0.016
Cerebrovascular disorders	5 (38.5 %)	10 (34.5 %)	0.813
Chronic pulmonary diseases	4 (30.8 %)	5 (17.2 %)	0.382
COPD	2 (15.4 %)	2 (6.9 %)	0.469
Bronchiectasis/NTM	1 (7.7 %)	5 (17.2 %)	0.370
Interstitial lung diseases	1 (7.7 %)	1 (3.4 %)	0.621
Diabetes mellitus	4 (30.8 %)	3 (10.3 %)	0.178
Dementia	4 (30.8 %)	6 (20.7 %)	0.519
Heart diseases	4 (30.8 %)	5 (17.2 %)	0.382
Hepatic diseases	1 (7.7 %)	1 (3.4 %)	0.621
Renal diseases	1 (7.7 %)	4 (13.8 %)	0.550
Hematology/Collagen-vascular diseases	1 (7.7 %)	4 (13.8 %)	0.550
ECOG performance status 3-4	75.0 % (9/12)	60.0 % (15/25)	0.371
Use of Glucocorticoid**/Immunosuppresant	1 (7.7 %)	5 (17.2 %)	0.37
Use of gastric tube	1 (7.7 %)	2 (6.9 %)	0.931
Histories/Risks of aspiration	8 (61.5 %)	15 (51.7 %)	0.568
Antibiotic therapy in the preceding 90 days	5 (38.5 %)	7 (24.1 %)	0.387
History of MRSA detection in the preceding 90 days	4 (30.8 %)	5 (17.2 %)	0.382
Type of pneumonia			
CAP	0 (0.0 %)	2 (6.9 %)	0.161
HCAP	2 (15.4 %)	15 (51.7 %)	0.015
HAP	11 (84.6 %)	12 (41.3 %)	0.099
Radiologic findings of Chest CT*			
Consolidation	7 (63.6 %)	12 (41.4 %)	0.474
Bronchopneumonia	1 (9.1 %)	9 (31.0 %))	0.052
Complicated with cavitation/abscess formation	0 (0.0 %)	0 (0.0 %)	–
Complicated with atelectasis	0 (0.0 %)	3 (10.3 %)	0.083
Centrilobular nodules (DPB-like)	0 (0.0 %)	0 (0.0 %)	–
Diffuse alveolar shadow (ARDS-like)	2 (18.2 %)	0 (0.0 %)	0.165
Bronchiectasis	1 (9.1 %)	6 (20.7 %)	0.239
Parapneumonic pleural effusion	5 (45.5 %)	3 (10.3 %)	0.082

*SD* standard deviation, *COPD* chronic obstractive pulmonary disease, *NTM* nontuberculous mycobacterial infection, *MRSA* methicillin-resistant *Staphylococcus aureus*, *ECOG* Eastern Cooperative Oncology Group, *CAP* community-acquired pneumonia, *HCAP* helathcare-associated pneumonia, *HAP* hospital-acquired pneumonia, *CT* computed tomography, *DPB* diffuse pulmonary bronchiolitis, *ARDS* acute respiratory distress syndrome

*includes diplicates, **corresponds to prednisolone 5 mg daily or greater

### Comparison between bacterial cultivation and the clone library method of 16S rRNA gene sequencing analysis

Table 2 shows the comparison of the results of the bacterial culture and bacterial floral analysis using the 16S rRNA gene with the clinical course of the patients. Cultivation results demonstrated that MRSA were isolated in all 19 patients in whom sputum culture was performed, and 37 of 41 (90.5 %) patients excluding No. 33 (BALF culture was not analyzed) showed positive culture results of MRSA using BALF samples. The molecular method detected the *S. aureus* phylotype in 22 patients, whereas 20 patients showed no *S. aureus* phylotypes in the BALF samples (Table 2). In Group A, *S. aureus* was detected in 69.2 % (9 of 13, Cases 1–9) of the patients by both cultivation methods and the molecular method, and the molecular method demonstrated that the *S. aureus* phylotype was predominant in 38.5 % (5 of 13, Cases 1–5) of the BALF specimens (Table 2). In 8 of 13 (61.5 %) Group A patients where *S. aureus* was not the predominant phylotype (Cases 6–13), *S. aureus* comprised a minor population (percentage of clones ≤ 10 %). In these 8 patients (Cases 6–13), no discrepancies were observed between the most occupied bacterial phylotypes by the molecular method and the clinical outcomes after antibiotic therapy. All of the patients with poor clinical outcomes in Group A (5 of 13, Cases 1, 4, 6, 10, 11) had obvious complicated poor prognostic factors, such as asphyxiation due to tracheobronchial secretion, exacerbation of heart failure and aspergillosis.

**Table 2:** Comparison of Bacteria Between Conventional Cultivation Methods and 16S rDNA Sequencing Analysis in the Bacterial Infection Group

Age		Pneumonia type	Sputum	BALF			Prior antibiotics	Treatments	Clinical outcome
			Culture	Culture	Clone library analysis				
					Predominant phylotype, %	*S. aureus*			
GROUP A									
1	72	HAP	*not analyzed*	*MRSA*	*Staphylococcus aureus 97.5 %*	*97.5 %*	ABK	VCM	ineffective
2	70	VAP	*not analyzed*	*MRSA*	*Staphylococcus aureus 91.8 %*	*91.8 %*	None	TEIC	effective
3	78	HAP	*MRSA*	*MRSA*	*Staphylococcus aureus 91.0 %*	*91.0 %*	None	ABK	effective
4	77	HCAP	*MRSA*	*MRSA*	*Staphylococcus aureus 53.1 %*	*51.8 %*	IPM/CS MINO	BIPM, PZFX + CLDM, CZOP + ABK	ineffective
5	65	HAP	*not analyzed*	*MRSA*	*Staphylococcus aureus 50.0 %*	*50.0 %*	MEPM	MEPM+VCM	effective
6	76	HAP	*not analyzed*	*MRSA*	*Corynebacterium simulans 41.9 %*	*8.1 %*	None	VCM	ineffective
7	61	HAP	*S. pneumoniae MRSA*	*MRSA*	*Haemophilus influenzae 35.3 %*	*3.5 %*	MEPM	MEPM + VCM	effective
8	87	HAP	*MRSA*	*MRSA*	*Corynebacterium spp. 97.8 %*	*2.2 %*	SBT/ABPC	TEIC	effective
9	66	HAP	*not analyzed*	*H. influenzae, MRSA*	*Haemophilus influenzae 84.0 %*	*1.1 %*	None	IPM/CS + VCM SBT/ABPC	effective
10	82	VAP	*not analyzed*	*MRSA P. aeruginosa*	*Pseudomonas aeruginosa 94.6 %*	*0.0 %*	IPM/CS	PZFX+VCM	ineffective
11	61	HCAP	*not analyzed*	*MRSA, Aspergillus fumigatus*	*Streptococcus spp. 90.7 %*	*0.0 %*	DRPM	DPPM PZFX + CLDM/L-AMB + VCM/TAZ/PIPC	ineffective
12	91	HAP	*MRSA*	*MRSA*	*Streptococcus oralis 58.5 %*	*0.0 %*	MEPM	LZD	effective
13	87	HAP	*not analyzed*	*MRSA, Neisseria*	*Neisseria perflava 95.5 %*	*0.0 %*	TEIC	TEIC, AMK	effective
GROUP B									
14	81	HAP	*not analyzed*	*MRSA*	*Staphylococcus aureus 100 %*	*100.0 %*	None	DRPM	effective
15	21	VAP	*not analyzed*	*MRSA*	*Staphylococcus aureus 88.6 %*	*88.6 %*	None	MEPM	effective
16	73	CAP	*not analyzed*	*MRSA*	*Staphylococcus aureus 60.8 %*	*60.8 %*	None	GRX	effective
17	76	HCAP	*MRSA P. aeruginosa*	*MSSA*	*Staphylococcus aureus 57.1 %*	*57.1 %*	None	TAZ/PIPC	ineffective
18	81	HCAP	*MRSA P. aeruginosa*	*no growth*	*Staphylococcus aureus 55.4 %*	*55.4 %*	None	CZOP + CLDM	effective
19	62	CAP	*MRSA*	*MRSA*	*Staphylococcus aureus 48.7 %*	*48.7 %*	FQ	CPFX	effective
20	80	HAP	*MRSA*	*MRSA*	*Streptococcus intermedius 56.5 %*	*40.6 %*	None	DRPM	effective
21	76	HAP	*MRSA*	*MRSA*	*Corynebacterium spp. 25.3 %*	*18.4 %*	TAZ/PIPC	SBT/ABPC	effective
22	22	HAP	*not analyzed*	*MRSA, A. baumannii*	*Neisseria elongata 81.2 %*	*15.1 %*	TEIC	IPM/CS	effective
23	81	HCAP	*MRSA*	*MRSA*	*Streptococcus spp. 46.7 %*	*12.0 %*	None	LVFX	effective
24	85	HAP	*not analyzed*	*MRSA*	*Streptococcus oralis/mitis 37.3 %*	*8.0 %*	CPFX	LVFX	effective
25	83	HCAP	*MRSA, E. coli*	*MRSA, E. coli*	*Moraxella catarrhalis 69.2 %*	*7.7 %*	None	TAZ/PIPC	effective
26	76	HAP	*not analyzed*	*K. pneumoniae, MRSA, Proteus mirabilis*	*Corynebacterium simulans 58.4 %*	*1.3 %*	None	TAZ/PIPC	effective
27	85	HAP	*MRSA*	*MRSA*	*Fusobacterium nucleatum 55.7 %*	*0.0 %*	None	LVFX	effective
28	61	HCAP	*not analyzed*	*MRSA, B. cepacia, F. mortiferum*	*Rothia spp. 45.2 %*	*0.0 %*	None	MEPM	effective
29	45	HAP	*not analyzed*	*S. maltophilia, MRSA*	*Enterococcus hirae 25.8 %*	*0.0 %*	None	MEPM	effective
30	74	HCAP	*P. aeruginosa MRSA*	*P. aeruginosa, MRSA*	*Streptococcus salivarius 43.0 %*	*0.0 %*	None	MEPM	effective
31	80	HCAP	*not analyzed*	*MRSA*	*Streptococcus spp. 98.9 %*	*0.0 %*	Unknown	LVFX	effective
32	80	HCAP	*not analyzed*	*MRSA*	*Streptococcus spp. 97.4 %*	*0.0 %*	Unknown	LVFX	effective
33	98	HCAP	*MRSA*	*not analyzed*	*Streptococcus spp. 78.8 %*	*0.0 %*	Unknown	TAZ/PIPC	effective
34	80	HCAP	*MRSA*	*oral bacteria*	*Neisseria spp. 55.0 %*	*0.0 %*	LVFX	MEPM	effective
35	82	HCAP	*not analyzed*	*P. aeruginosa, MRSA, Streptococcus*	*Streptococcus oralis/mitis 70.7 %*	*0.0 %*	Unknown	SBT/ABPC	effective
36	64	HCAP	*not analyzed*	*P. aeruginosa, MRSA*	*Pseudomonas aeruginosa 97.4 %*	*0.0 %*	None	TAZ/PIPC + LVFX	effective
37	86	HCAP	*MRSA*	*K. pneumoniae, MRSA*	*Lactobacillus spp. 51.1 %*	*0.0 %*	None	TAZ/PIPC	effective
38	80	HCAP	*E. coli, MRSA*	*oral bacteria*	*Streptococcus spp. 45.2 %*	*0.0 %*	None	TAZ/PIPC	effective
39	93	HCAP	*not analyzed*	*MRSA, oral bacteria*	*Corynebacterium spp. 94.3 %*	*0.0 %*	None	LVFX	effective
40	81	HAP	*not analyzed*	*MRSA, E. coli*	*Haemophilus influenzae 34.5 %*	*0.0 %*	None	TAZ/PIPC	effective
41	73	HAP	*not analyzed*	*Enterobacter cloacae, MRSA*	*Enterobacter asburiae 70.0 %*	*0.0 %*	None	DRPM	effective
42	74	HAP	*MRSA*	*Corynebacterium, MRSA*	*Corynebacterium simulans 98.9 %*	*0.0 %*	TAZ/PIPC	TAZ/PIPC	effective

*Abbreviations*: *CAP* community-acquired pneumonia, healthcare-associated pneumonia, *HAP* hospital-acquired pneumonia, *VAP* ventilator-associated pneumonia, *MRSA* methicillin-resistant *Staphylococcus aureus*, *BALF* bronchoalveolar lavage fluid, *ABK* arbekacin, *VCM* vancomycin, *TEIC* teicoplanin, *LZD* linezolid, *IPM/CS* imipenem/cilastatin, *MEPM* meropenem, *DRPM* doripenem, *BIPM* biapenem, *CZOP* cefozopran, *SBT/ABPC* sulbactam/ampicillin, *TAZ/PIPC* tazobactam/piperacillin, *CPFX* ciprofloxacin, *LVFX* levofloxacin, *GRNX* garenoxacin, *MINO* minomycin, *CLDM* clindamycin, *L-AMB* liposomal amphotericin B, *NA* not applicable

In Group B, 96.5 % of patients (28 of 29, Cases 14–16, 18–42) showed good clinical outcomes without anti-MRSA antimicrobials; one patient (Case 17) died because of asphyxiation due to tracheobronchial secretion. The *S. aureus* phylotype was a minor population (percentage of clones ≤10 %) or undetectable (0 %) in 10.7 % (3 of 28, Cases 24–26) and 57.1 % (16 of 28, Cases 27–42), respectively, of the patients in Group B who had good clinical outcomes. In addition, 5 of these 28 patients (Cases 14–18) showed that the *S. aureus* phylotype was the most detected phylotype, including one patient (Case 14) with 100 % of the percentage of clones of *S. aureus* phylotype.

### Correlation of the percentage of clones of *S. aureus* phylotype and risk factors of MRSA pneumonia

Table 3 shows the relationship between the percentage of clones of the *S. aureus* phylotype and the risk factors of MRSA pneumonia among all 42 patients. Gastrostomy or nasogastric tube feeding was significantly negatively correlated with the percentage of clones of *S. aureus* phylotype using the molecular method (Table 3).

**Table 3:** Comparison of Bacteria Between Conventional Cultivation Methods and 16S rDNA Sequencing Analysis in the Bacterial Infection Group

Risk factors (Positive/Negative)**	The percentage of clones of MRSA phylotype (%)		
	Positive	Negative	*P*-value
Use of Glucocorticoid**/Immunosuppressant (6/36)	40.2 ± 41.4	19.0 ± 28.3	0.305
Histories/Risks of aspiration (23/19)	18.7 ± 33.2	25.2 ± 29.2	0.422
Antibiotic therapy in the preceding 90 days (12/30)	30.1 ± 40.7	17.5 ± 25.7	0.391
History of pathogens detection in the preceding 90 days* (9/30)	38.2 ± 35.1	15.4 ± 28.9	0.105

*Unknown data in three cases, **“Positive” and “Negative” indicate the number of patients with or without each risk factor, respectively.

*Abbreviations*: *MRSA* methicillin-resistant *Staphylococcus aureus*

## Discussion

We analyzed the cultivation results and bacterial phylotypes according to the molecular method using BALF samples in patients with MRSA cultured from respiratory samples, and interestingly, no clones of *S. aureus* were detected in the BALF samples in 47.6 % (20/42) of these patients. Most of the patients (*n* = 28 of 29; 96.5 %) treated without anti-MRSA antimicrobials (Group B) showed favorable clinical outcomes despite the cultivation of MRSA, and these 28 patients were suspected to have non-MRSA pneumonia; the cultured MRSA from the respiratory samples might have been due to colonization in the respiratory tract. In addition, the *S. aureus* phylotype was only a minor population (the percentage of clones ≤ 10 %) in 67.9 % (19/28) of these 28 patients in Group B according to the molecular method, which was compatible with their clinical courses. Several previous reports have described that MRSA is occasionally a non-causative pathogen of pneumonia in some patients [23–26] even when MRSA is cultured from sputum samples, and our results suggest that even when MRSA was cultured using samples obtained from the lower respiratory tract, MRSA was clinically considered not to be a causative agent in more than two-thirds of these patients.

A similar report by Nagaoka et al. showed that approximately half (51.4 %, 36/70) of the patients were considered to have true MRSA pneumonia when hospital-acquired MRSA pneumonia was defined according to the positive responses and/or clinical demand of anti-MRSA agents with a positive culture of MRSA and detection of clustered Gram-positive cocci within polymorphonuclear cells in the respiratory samples, such as BALF or transthoracic aspiration [11]. Moreover, at least 66.7 % (28/42) of the patients were possibly considered to have MRSA colonization, and MRSA was considered to be a causative pathogen in 33.3 % (14/42) of the patients in this study. These data suggest that it remains clinically controversial whether or not MRSA is a true causative pathogen of pneumonia, even in patients with MRSA cultured from the lower respiratory samples, and the ratio of true MRSA pneumonia in these patients might be lower than previously believed.

Five of 28 (17.9 %) patients in Group B who showed good clinical outcomes without anti-MRSA agents demonstrated that the *S. aureus* phylotype was predominant among the detected bacterial phylotypes in the samples, which may be inconsistent with the colonization of MRSA. The molecular method we used could not evaluate drug resistance, and a differentiation between MRSA and methicillin-susceptible *S. aureus* (MSSA) was not possible in this retrospective study. Spontaneous remission of MRSA pneumonia is another potential explanation. In addition, there are presently no criteria to differentiate causative pathogens using the ratio of bacterial phylotypes in the samples, thus careful discretion is necessary to interpret these data, and further studies are needed to elucidate this issue.

Several guidelines [4, 6–8] and clinical trials [11] have described the risk factors of MRSA pneumonia. According to a report by Nagaoka et al. [11] that used a multiple regression analysis for the risk factors of MRSA, a past history of head and neck, esophageal or stomach surgery (odds ratio (OR) 8.63), radiological findings of other than lobar pneumonia (OR 10.2), severity of pneumonia with the Pneumonia Patient Outcomes Research Team (PORT) score 5 (OR 5.23), more than 10^6^ CFU/ml of MRSA using a quantitative culture (OR 12.8), and a single cultivation of MRSA (OR 19.9) were significantly correlated with MRSA pneumonia. More HCAP patients were observed in Group B than in Group A, however, no other factors were significantly different between Groups A and B in this study. When considering the percentage of clones of the *S. aureus* phylotype in BALF samples according to each different risk factor in this study, only the “use of gastric tube feeding” was inversely correlated with the percentage of clones of *S. aureus* (Table 3), suggesting that such condition may be a clue to avoid an abuse of anti-MRSA agents.

The analysis using the 16S rRNA gene can detect only bacterial DNA, and does not equally indicate that the detected bacterial phylotype causes bacterial infection. Therefore, we have been investigating and validating this molecular method in several diseases to compare this molecular method with the results of cultivation methods in several settings [12, 13]. Further investigations for validating this method should be performed.

### Study limitation

There are several limitations associated with the present study. First, the universal primers we used for the molecular analysis could not amplify all of the bacterial 16S rRNA genes. The primers we used cover approximately 92 % of the registered bacterial species in the Ribosomal Database Project II database, however, the remaining undetectable bacteria with these primers included no causative pathogens that have been reported in humans [19]. Second, approximately 100 clones per each clone library were analyzed, meaning that bacterial 16S rRNA gene sequences present at less than 1 % of each sample may not be detectable using this method. Third, this study was retrospective, and anti-MRSA agents were administered with no particular criteria. Fourth, a quantitative culture and evaluation of neutrophil phagocytosis of the organisms were not performed. Fifth, study population was relatively small and elderly patients were mostly included in HCAP or HAP patients, and only two CAP patients were included. Further investigations should be considered to elucidate the data in younger population.

## Conclusion

We evaluated the clinical course and the ratios of bacterial phylotypes in BALF specimens using the clone library method and conventional cultivation methods in patients with MRSA detected by cultivation from respiratory samples. The results of this study demonstrated that these patients were heterogeneous, and approximately two-thirds of these patients might be considered to have MRSA colonization or non-MRSA pneumonia. In addition, the results of the cultivation-independent molecular method we used indicated that the detection of MRSA by cultivation methods may not correctly reflect the pathogenicity of MRSA in patients with pneumonia. Further prospective studies are necessary to elucidate the pathogenicity of MRSA in pneumonia.

### Ethics approval and consent to participate

This study was approved by the Human and Animal Ethics Review Committee of the University of Occupational and Environmental Health, Japan (No.09-118), and all patients provided their written informed consent.

## Availability of data and materials

We declare that the data supporting the conclusions of this article are fully described in the article.

## Abbreviations

ATS: American Thoracic Society
BALF: bronchoalveolar lavage fluid
BLAST: the basic local alignment search tool
CAP: community-acquired pneumonia
CFU: colony forming units
CT: computed tomography
HAP: hospital-acquired pneumonia
HCAP: healthcare-associated pneumonia
IDSA: Infectious Diseases Society of America
MRSA: methicillin-resistant *Staphylococcus aureus*
MSSA: methicillin-susceptible *S. aureus*
NHCAP: nursing and healthcare-associated pneumonia
OR: odds ratio
PCR: polymerase chain reaction
PORT: Pneumonia Patient Outcomes Research Team
VAP: ventilator-associated pneumonia
WBC: white blood cell
